# Effect of Sodium and Chloride Binding on a Lecithin Bilayer. A Molecular Dynamics Study

**DOI:** 10.3390/membranes7010005

**Published:** 2017-01-25

**Authors:** Maria M. Reif, Christopher Kallies, Volker Knecht

**Affiliations:** 1 Physics Department (T38), Technische Universität München, James-Franck-Str. 1, Garching 85748, Germany; maria.reif@tum.de; 2 Institute of Physics, Albert-Ludwigs-Universität Freiburg, Hermann-Herder-Str. 3, Freiburg 79104, Germany; christopher_kallies@web.de; 3 Freiburg Centre for Interactive Materials and Bioinspired Technologies, Georges-Köhler-Allee 105, Freiburg 79110, Germany

**Keywords:** molecular dynamics, POPC bilayer, salt effects, lipid force field, ion force field, sodium chloride

## Abstract

The effect of ion binding on the structural, mechanical, dynamic and electrostatic properties of a 1-palmitoyl-2-oleoyl-*sn*-glycero-3-phosphocholine (POPC) bilayer in a 0.5 M aqueous NaCl solution is investigated using classical atomistic molecular dynamics simulation with different force-field descriptions for ion-ion and ion-lipid interactions. Most importantly, the repulsive Lennard–Jones parameters for the latter were modified, such that approximately similar binding of cations and anions to the lipid membrane is achieved. This was done to qualitatively improve the apparent ion-lipid binding constants obtained from simulations with the original force field (Berger lipids and GROMOS87 ions in combination with the SPC water model) in comparison to experimental data. Furthermore, various parameters characterizing membrane structure, elasticity, order and dynamics are analyzed. It is found that ion binding as observed in simulations involving the modified in comparison to the original force-field description leads to: (i) a smaller salt-induced change in the area per lipid, which is in closer agreement with the experiment; (ii) a decrease in the area compressibility and bilayer thickness to values comparable to a bilayer in pure water; (iii) lipid deuterium order parameters and lipid diffusion coefficients on nanosecond timescales that are very similar to the values for a membrane in pure water. In general, salt effects on the structural properties of a POPC bilayer in an aqueous sodium-chloride solution appear to be reproduced reasonably well by the new force-field description. An analysis of membrane-membrane disjoining pressure suggests that the smaller salt-induced change in area per lipid induced by the new force-field description is not due to the alteration of membrane-associated net charge, but must rather be understood as a consequence of ion-specific effects on the arrangement of lipid molecules.

## 1. Introduction

The presence of ions crucially influences membranes in terms of structural and mechanical properties, dynamics, thermodynamic stability and biological function [1,2,3,4,5,6,7]. Understanding the molecular mechanisms underlying membrane structure and mechanics is important not only because of the biological relevance of membranes in aqueous electrolyte solutions, but also because the structural and mechanical characteristics of membranes will, in the future, be of increasing interest in the development of synthetic materials or engineered tissues [8,9].

The strength of ion-membrane binding is governed by the equilibrium between solvation and the membrane-interaction of the ions. Intuitively, electrostatic interactions are a key determinant of this equilibrium, which is why, at first glance, considerable differences in ion-membrane binding may be expected depending on the ion valency and the membrane charge. Divalent ions show stronger binding than monovalent ones, e.g., the (intrinsic) binding constants of Na${}^{+}$, Cl${}^{-}$ and Ca${}^{2+}$ to zwitterionic 1,2-dipalmitoyl-*sn*-glycero-3-phosphatidylcholine (DPPC) liposomes were measured at 25 °C as 0.25 M${}^{-1}$, 0.28 M${}^{-1}$ and 37 M${}^{-1}$, respectively [10]. For comparison, the intrinsic binding constant at 25 °C of Na${}^{+}$ to anionic phosphatidylserine-containing vesicles was suggested to be 0.6 M${}^{-1}$ [11]. This illustrates the seemingly important role of electrostatic interactions. Considering the release of tightly-bound solvation-shell water molecules that accompanies the desolvation of a membrane-associated ion, entropy gain may also be seen as a main driving force for the binding of ions to membranes [5]. The two mechanisms (the role of electrostatic interactions and entropy gain of released water) are, however, intimately connected, because the Coulomb interaction energy (between two charged species in water, expressing the solvent degrees of freedom via the dielectric constant of the solvent) can, due to the temperature dependence of the solvent relative dielectric permittivity, be interpreted as a free energy [12], with entropic and enthalpic components per unit charge of −TΔS=1.3 V and ΔH=−0.3 V, respectively. Moreover, one may expect that the ionic strength of the electrolyte solution or the presence of other salts can affect ion binding constants, because other salts can, for instance, decrease the hydration of an ion in the electrolyte and, thus, enhance its association with the membrane.

Physical insight into the thermodynamics of ion-membrane association can be obtained experimentally via radiotracing of radioactive ion isotopes [13], measurement of membrane electrophoretic mobilities [10,11,14], isothermal titration calorimetry [5,14] or via computer experiments, e.g., through molecular dynamics (MD) simulation [14,15]. Due to the heterogeneous nature of the membrane (biological membranes are composed of a variety of lipids and proteins) and its environment (electrolyte solution), as well as the physical complexity of the forces at play (long-range electrostatic, short-range van der Waals interactions), neither a theoretical understanding, nor a predictive theoretical description of this equilibrium are straightforward. It was pointed out before [16] that current models of specific ion effects are not sufficient to explain the interaction of chaotropic anions and kosmotropic cations with lipid membranes, i.e., the fact that anions and cations show opposite Hofmeister-series behavior, which hints at different binding mechanisms for the two types of ions. For zwitterionic lecithin (phosphatidylcholine) lipids, ion-lipid association can, to a certain extent, be explained in terms of the empirical law of matching water affinities [17], based on which Garcia-Celma et al. [16] interpreted cation binding to phosphate groups (both of kosmotropic nature) and anion binding to choline groups (both of chaotropic nature). However, this interpretation fails for other lipids, e.g., anionic surfactants [16]. Leontidis and Aroti [18] suggested that the different interaction behavior of cations and anions with the lipid membrane is drawn back to cations interacting directly with lipid headgroups, thus giving rise to density inhomogeneities through a clustering of lipids, whereas anions invade the created space of low lipid density. They also stressed the potential MD simulation has in unraveling ion-membrane interactions.

Numerous MD studies on ion-membrane interactions were performed in the past, e.g., focusing on the kinetics, stoichiometry and structural aspects of ion-lipid binding [19,20,21,22,23] and the specific ion effects thereof [24,25,26,27]. Partly, the results have been found to depend strongly on the employed force field [22], and it was emphasized that careful force-field optimization might be required to achieve simulation results in agreement with experimental findings [14]. However, such a force-field recalibration is highly intricate as its aims, namely an accurate representation of both ion-ion, the ion-membrane functional group and ion-water interactions, might be irreconcilable with the approximations made by current “simple” force-field descriptions. In other words, it might be required to circumvent combination rules for Lennard–Jones parameters or/and account (explicitly) for polarization effects. Nevertheless, MD simulation is a valuable tool to investigate ion-membrane systems. Besides providing information about the thermodynamics of ion-membrane binding, it also offers atom-level insight concerning the structure, mechanics, order and dynamics of membranes.

Phospholipid membranes can exist in various alternative phases. The biologically most relevant phase is the liquid-crystalline phase, which is fluid-like, presenting the lipid chains in rather disordered configurations. The flexible nature of the membrane renders it susceptible to several types of deformation that may occur at room temperature and atmospheric pressure. The four most common types of membrane deformation include [28] changes of membrane curvature, area, thickness and deformation due to shear forces. For a phospholipid bilayer, the most important deformation at room temperature is a change of curvature, i.e., bending. The associated bending rigidity is around 10–20 $k_{B}T$, where $k_{B}$ is Boltzmann’s constant and *T* the absolute temperature. The energetic cost of area changes is higher, Hooke’s law force constant for an area change being around 55–70 $k_{B}T\cdot$nm${}^{-2}$. Stretching or compressing the membrane along its normal has a similar Hooke’s law force constant of around 60 $k_{B}T\cdot$nm${}^{-2}$. Finally, shearing is not of relevance for fluid-like membranes, such as a membrane in the liquid-crystal phase, but may occur when membranes are attached to solid structures.

When lipid bilayers are immersed in aqueous electrolyte solutions in comparison to pure water, MD simulations evidence a reduction of membrane fluidity (i.e., of lateral lipid diffusion), a reduction of the area per lipid and an increase in membrane bilayer thickness [14,19,23,29]. Similar findings were obtained experimentally [4], although the influence of salt concentration on some structural membrane properties appears to be more complex in the case of certain salts. For example, in the case of NaCl, the bilayer separation (thickness of the inter-membrane water layer) increases monotonously with salt concentration, whereas it decreases with increasing CaCl${}_{2}$ concentration [4]. Zimmermann et al. [7] analyzed the effect of different salts on membrane fluidity in terms of a relationship between membrane fluidity and membrane charge. It was observed that, although chaotropic anions and kosmotropic cations can influence fluidity in a similar fashion, they have a different impact on membrane charge. A simple explanation for the effect of ions on membrane fluidity might be that the decrease of fluidity is caused by dehydration of lipid molecules due to ions near the membrane-water interface [29].

In general, lipid order [4] and area compressibility [1] are found to increase upon the addition of salt. Concomitant with the increase of order, the elasticity of the bilayer decreases [4]. A number of experimental studies on the bending stiffness of lipid membranes were performed [30,31,32], illustrating, e.g., the influence of the electrostatic characteristics of the membrane-water interface [30,32] or membrane-active compounds [31] on membrane mechanical properties. Membrane-water interfacial electrostatic properties are commonly described by the zeta potential, and it was found [30] that the relation between zeta potential and bending stiffness is qualitatively captured by the theoretical model of Helfrich and Winterhalter [33,34]. Thus, one can decipher how ion binding affects membrane mechanics via altering surface charge. Ion binding does not only influence the properties of the membrane-water interface, but also intramembrane electrostatic properties, such as the membrane dipole potential. However, supposedly, there is no immediate connection between the membrane dipole potential and membrane mechanical properties [30].

In MD simulations, surface charge is expected to be reflected in both physical (membrane stiffness) and artificial phenomena. As MD simulations of membrane-solvent systems are commonly performed under periodic boundary conditions, i.e., the separation of periodic bilayer copies (along the bilayer normal) is finite rather than infinite, membrane interfacial electrostatic properties translate into artificial repulsive forces between bilayer copies. The magnitude of the resulting disjoining pressure [35] is proportional to the membrane area compressibility and the inverse of the solvent layer thickness. Thus, it may be difficult to separate physical and artificial effects of the membrane surface charge induced by ion-membrane association. An intuitive reasoning suggests that surface charge correlates with inter-bilayer repulsion, hence increasing inter-bilayer separation (i.e., the thickness of the solvent layer) and (due to solvent incompressibility) decreasing area per lipid.

In the present study, MD simulations are performed to investigate the above reasoning about salt effects on membranes. In particular, an existing ion-lipid force field is recalibrated to achieve similar binding affinities for Na${}^{+}$ and Cl${}^{-}$ ions to a 1-palmitoyl-2-oleoyl-*sn*-glycero-3-phosphocholine (POPC) bilayer in an aqueous approximately 0.5 M NaCl solution. It is studied how membrane properties, as well as electrostatic interfacial properties are altered. The goal is to investigate whether a correct description of ion-membrane binding behavior reproduces experimental trends concerning salt effects on membrane structure, mechanics, order and dynamics. The paper is organized as follows. Section 2 gives a brief outline of the employed biophysical theory about membrane-ion interactions, membrane-solvent interfacial electrostatics and inter-bilayer electrostatic forces. Section 4 provides computational details about the MD simulations. Corresponding results are reported and discussed in Section 3. Finally, Section 5 summarizes the main conclusions.

## 2. Theory

### 2.1. Ion-Lipid Binding Constants

Assuming a membrane in a monovalent salt and a 1:1-stoichiometric association of an ionic species *I* with lipid molecules, one can define an apparent binding constant $K_{\mathrm{app}}\left(I\right)$ as:

(1) $$K_{\mathrm{app}}\left(I\right)=\frac{N_{b}\left(I\right)}{\left(N_{L}-N_{b}\left(I\right)\right){\bar{c}}_{\mathrm{blk}}}={\left({\bar{c}}_{\mathrm{blk}}\right)}^{-1}\frac{\alpha (I)}{1-\alpha (I)},$$

where the law of mass action was used, with $\alpha \left(I\right)=\frac{N_{b}\left(I\right)}{N_{L}}$ denoting the fraction of the number of ions bound to lipid molecules ($N_{b}$) and the total number of lipids ($N_{L}$) and ${\bar{c}}_{\mathrm{blk}}$ denoting the mean salt concentration in the bulk [15,36]. Equation (1) captures an association equilibrium via counting concentrations of complexes and free components, i.e., straightforward application of the law of mass action. It implicitly assumes that the ratio of free and complexed species is representative of a macroscopic system in the sense that it does not suffer from the microscopic-system artifact encountered when determining a binding constant based on association frequency [37].

The apparent binding constant characterizes the attraction of an ion to the lipid membrane due to the ion-membrane affinity intrinsic to the nature of the formed ion-lipid complex, but also due to electrostatic forces that arise solely on the basis of the membrane zeta potential, Δζ. The latter contribution can be removed through the corresponding Boltzmann factor $\exp[-\beta q_{I}\Delta \zeta ]$, where $q_{I}$ is the ion charge and $\beta ={\left(k_{B}T\right)}^{-1}$, leading to the intrinsic binding constant:

(2) $$K_{\mathrm{int}}\left(I\right)=K_{\mathrm{app}}\left(I\right)\exp\left[\beta q_{I}\Delta \zeta \right].$$

Note that for vanishing surface charge, the zeta potential evaluates to zero, and therefore, $K_{\mathrm{app}}\left(I\right)=K_{\mathrm{int}}\left(I\right)$.

### 2.2. Finite-Size Effects

When lipid membranes are simulated under periodic boundary conditions, structural parameters, such as the area per lipid, are influenced by the periodic membrane copies along the bilayer normal. Due to their long-range nature, this finite-size artifact is predominantly mediated by electrostatic interactions between the surface charges of the bilayers. Denoting the area per lipid pertaining to infinite bilayer separation (i.e., infinite *z*-dimension of the simulated system) and zero surface tension with $a_{L,o,\infty }$ and that pertaining to the simulated system of finite *z*-dimension with $a_{L,o}$, the free energy associated with the change in area per lipid from $a_{L,o}$ to $a_{L,o,\infty }$ is:

(3) $$\Delta G^{\mathrm{FS}}=\kappa_{A}\frac{{\left(a_{L,o,\infty }-a_{L,o}\right)}^{2}}{2a_{L,o}}.$$

The area compressibility $\kappa_{A}$ in Equation (3) is given by:

(4) $$\kappa_{A}=\frac{\beta^{-1}a_{L,o}}{\left(N_{L}/2\right)\delta_{a_{L,o}}^{2}},$$

where $\delta_{a_{L,o}}^{2}$ is the mean square fluctuation of the area per lipid. In Equation (3), it is assumed that $\kappa_{A}$ does not change upon increasing the bilayer separation to infinity. The area per lipid at the infinite bilayer separation may be expressed in terms of the disjoining pressure [38]:

(5) $$P_{\mathrm{dis}}=\kappa_{A}d_{w}^{-1}\left(1-a_{L,o}^{-1}a_{L,o,\infty }\right),$$

where $d_{w}$ is the water layer thickness. Setting $P_{\mathrm{dis}}$ equal to the disjoining pressure present in an MD simulation, i.e., [38]:

(6) $$P_{\mathrm{MD}}=\kappa_{A}d_{w}^{-1}\left(1-a_{L,o}^{-1}a_{L}\right),$$

allows solving Equation (5) for $a_{L,o,\infty }$. Comparison of $a_{L,o,\infty }$ with the actual area per lipid observed during the simulation gives an estimate of the magnitude of finite-size effects on the simulated membrane structure.

### 2.3. Inter-Bilayer Forces

The disjoining pressure given by Equation (6) may also be used to establish a connection between membrane surface charge and membrane structural parameters (thickness and area). It characterizes the repulsion between periodic bilayer copies due to electrostatic forces between the associated surface charges. Comparing it with the theoretically-expected repulsion, captured here by the theoretically-expected disjoining pressure [35]:

(7) $$P_{\mathrm{TH}}=\beta^{-1}\left(\rho_{{\mathrm{Na}}^{+}}\left(z_{m}\right)+\rho_{{\mathrm{Cl}}^{-}}\left(z_{m}\right)-2{\left(\rho_{{\mathrm{Na}}^{+}}\left(z_{m}\right)\rho_{{\mathrm{Cl}}^{-}}\left(z_{m}\right)\right)}^{1/2}\right),$$

where $\rho_{{\mathrm{Na}}^{+}}\left(z_{m}\right)$ and $\rho_{{\mathrm{Cl}}^{-}}\left(z_{m}\right)$ are sodium and chloride ion number densities in the center of the water layer, gives insight into the mechanism underlying area and thickness alteration of the membrane, i.e., whether this alteration is really related to the change in surface charge that occurs when the membrane is immersed in an electrolyte solution rather than in pure water.

### 2.4. Membrane Rigidity and Area Compressibility

Modeling a lipid bilayer membrane as an array of thin elastic shells and describing the shell deformations with elasticity theory [39], one can express the area compressibility $\kappa_{A}$ (Equation (17)) and bending stiffness $k_{c}$ (bending rigidity; Equation (18)) in terms of the two-dimensional Lamé constants *λ* and *μ*. If one assumes a vanishing shear modulus, one can then derive a relation between bending stiffness, area compressibility and membrane thickness, captured by a coefficient *B* [40]:

(8) $$B=k_{c}\kappa_{A}^{-1}D_{\mathrm{HH}}^{-2},$$

where $D_{\mathrm{HH}}$ is the bilayer thickness. It was seen that the magnitude of *B* is a measure for the extent of interdigitation present between the two bilayer leaflets [41]. A value B=1/48 is characteristic of membranes showing little or no interdigitation, i.e., leaflets that can slide past each other with little hindrance, whereas a larger value, B=1/12, indicates coupled leaflet motion due to interdigitation. Note that the quadratic dependence of $k_{c}$ on $D_{\mathrm{HH}}$ in Equation (8) derives from a very simple membrane model. A more involved description of the membrane accounting for conformational entropy of the fatty acid chains suggests $k_{c}∼D_{\mathrm{HH}}^{5/2}$ [42].

## 3. Results and Discussion

Based on the simulations described in Section 4.1, the original and modified force-field descriptions for ion-lipid interactions were compared in terms of their influence on structural properties (membrane area per lipid, area compressibility, bilayer thickness, water layer thickness, lipid order parameters), the thermodynamics of ion-membrane binding (apparent binding constants, coordination numbers), lipid diffusion coefficients, electrostatic potential variation along the bilayer normal and membrane rigidity.

In simulations with the modified force-field version, the area per lipid increases in comparison to the original force field by about 16 (S${}_{\mathrm{NaCl}}$(m1,wc)) or 14% (S${}_{\mathrm{NaCl}}$(m2,wc); Table 1). It slightly exceeds the area per lipid of the membrane patch in pure water (0.607 nm${}^{2}$), namely by about 0.02 and 0.01 nm${}^{2}$ in simulations S${}_{\mathrm{NaCl}}$(m1,wc) and S${}_{\mathrm{NaCl}}$(m2,wc), respectively (Table 1). The results obtained with the modified force-field version entailing enhanced ion binding are thus in closer agreement to experimental data [4]. The area per lipid in simulations with the Parrinello–Rahman barostat is overall similar to that observed in simulations with the weak-coupling barostat (differences of 0–0.009 nm${}^{2}$).

**Figure 1:**
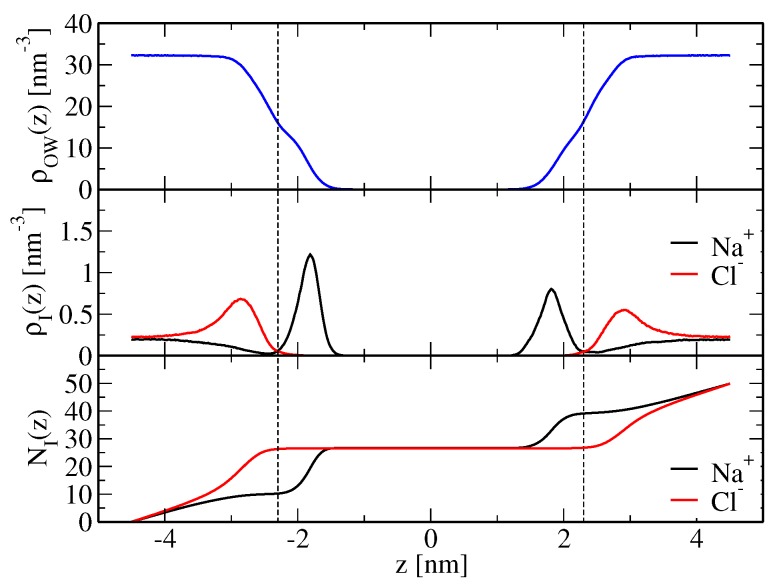
Profiles of water ($\rho_{\mathrm{OW}}\left(z\right)$) and ion ($\rho_{I}\left(z\right)$; I= Na${}^{+}$ or Cl${}^{-}$) number densities normal to the membrane, along with integrated ion density profiles giving the cumulative number of ions ($N_{I}\left(z\right)$; I= Na${}^{+}$ or Cl${}^{-}$), for simulation S${}_{\mathrm{NaCl}}$(o,wc), i.e., a simulation based on the unmodified force field (Table 7). The dashed vertical lines indicate *z*-values where the water oxygen number density evaluates to half of its bulk value, i.e., the location of the shear plane $z_{s}$ (Section 4.3.2). The corresponding apparent binding constants are reported in Table 2. Note that in this illustration, the lipid bilayer is centered.

As a consequence of lateral membrane expansion (along with solvent incompressibility), the thickness of the solvent layer $d_{w}$ decreases in simulations S${}_{\mathrm{NaCl}}$(m1,wc) and S${}_{\mathrm{NaCl}}$(m2,wc) compared to the simulation with the original force field (Table 1). In addition, the bilayer thickness $D_{\mathrm{HH}}$ decreases and basically adopts values observed for a membrane patch in pure water. For example, in simulations S${}_{\mathrm{NaCl}}$(m1,wc) and S${}_{\mathrm{NaCl}}$(m2,wc), $D_{\mathrm{HH}}$ evaluates to 3.86 and 3.95 nm, respectively, i.e., it is very similar to the thickness found in simulation $S_{\mathrm{wat}}$(wc), 3.98 nm, and smaller than that found in simulation S${}_{\mathrm{NaCl}}$(o,wc), 4.31 nm. Experimentally, the presence of aqueous NaCl leads to an increase in $D_{\mathrm{HH}}$ above NaCl concentrations of 0.6 M [4]. This increase in bilayer thickness is in qualitative agreement with simulation S${}_{\mathrm{NaCl}}$(o,wc), but not with the simulations involving the new force-field versions. In comparison to simulation S${}_{\mathrm{NaCl}}$(o,wc), the binding of approximately equal amounts of cations and anions (S${}_{\mathrm{NaCl}}$(m1,wc), S${}_{\mathrm{NaCl}}$(m2,wc)) induces a contraction of the membrane along the bilayer normal and a lateral expansion. Intuitively, one can try to explain this in terms of repulsion between charged bilayer copies as present in simulation S${}_{\mathrm{NaCl}}$(o,wc). Here, predominantly sodium ions bind below the membrane shear plane, whereas chloride ions are mainly located above the shear plane (Figure 1). Therefore, the membrane presents a net positive charge. An intuitive reasoning would suggest that the resulting electrostatic repulsion between periodic bilayer copies increases solvent layer thickness. Furthermore, due to solvent incompressibility, the xy-plane of the simulation box has to contract, which would be reflected in a decreased area per lipid (Table 1). However, an alternative mechanism is not based on electrostatic grounds, but ion-specific effects arising from direct ion-lipid interactions, i.e., the way in which ions bind to the lipid molecules may directly cause lateral compression or extension of the membrane. This is discussed in more detail below.

**Figure 2:**
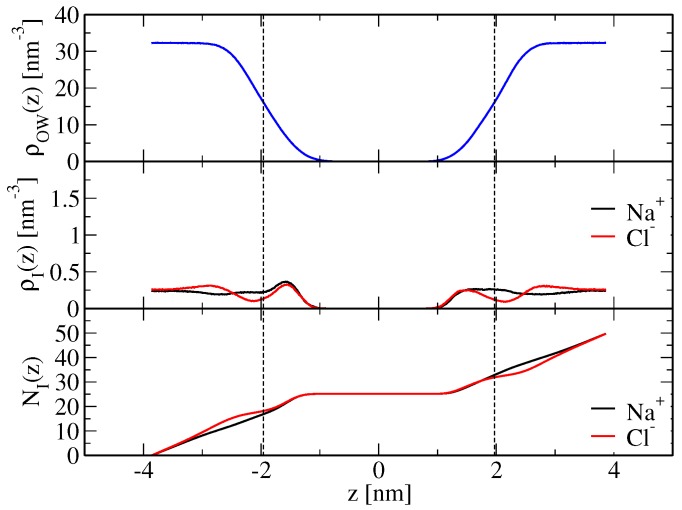
As Figure 1, but for simulation S${}_{\mathrm{NaCl}}$(m1,wc), i.e., a simulation based on a modified force field (Table 7). The corresponding apparent binding constants are reported in Table 2.

**Figure 3:**
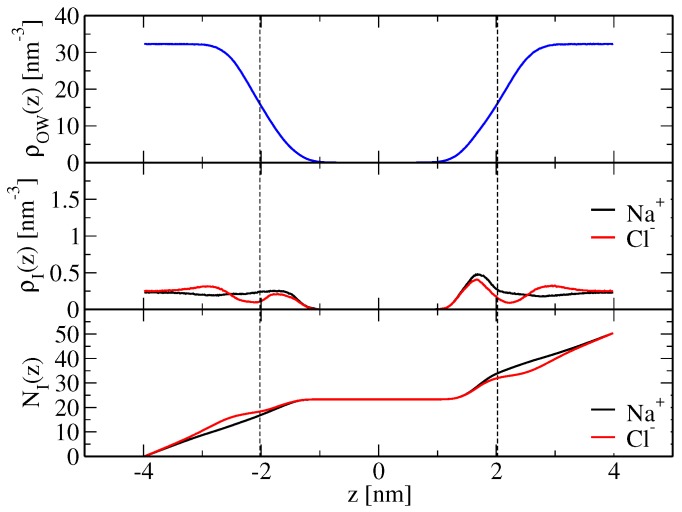
As Figure 1, but for simulation S${}_{\mathrm{NaCl}}$(m2,wc), i.e., a simulation based on a modified force field (Table 7). The corresponding apparent binding constants are reported in Table 2.

An increase of sodium-lipid and a decrease of chloride-lipid nonpolar repulsion, as achieved here by a scaling of corresponding Lennard–Jones $C_{12}$ parameters (Section 4.2), brings both cation and anion binding in better agreement with the experimental findings. Apparent ion-membrane binding constants from electrophoresis and isothermal titration measurements [14] are 0.437±0.046 M${}^{-1}$ for Na${}^{+}$ ions and 0.401±0.038 M${}^{-1}$ for Cl${}^{-}$ ions. They may be compared to the corresponding values from the simulations performed here, which are reported in Table 2 and were calculated according to Equation (21) based on the simulated ion density profiles along the bilayer normal, shown in Figure 2 and Figure 3 for the modified force-field versions m1 and m2, respectively. Sodium ion binding is overestimated by around a factor two in the original force field, i.e., $K_{\mathrm{app}}\;=\;0.87\;\pm \;0.16$ M${}^{-1}$, using the arithmetic mean of the binding constants pertaining to the two leaflets (0.71 and 1.02 M${}^{-1}$; Table 2), with the indicated error denoting the standard deviation. However, it is essentially in line with experimental data when the modified force fields m1 or m2 are used. The binding constants are $K_{\mathrm{app}}=0.35\pm 0.01$ and 0.39±0.11 M${}^{-1}$, respectively, again resulting from averaging over the two leaflets and using the standard deviation as an error estimate (Table 2). Chloride ion binding is significantly underestimated by the original force field ($K_{\mathrm{app}}=0.011\pm 0.002$ M${}^{-1}$). By virtue of decreased chloride-ion lipid-tail nonpolar repulsion, the modified force-field versions allow those ions to cross the membrane shear plane (Figure 2 and Figure 3), thus leading to an increase in their apparent binding constants ($K_{\mathrm{app}}=0.30\pm 0.01$ and 0.30±0.09 M${}^{-1}$ in simulations S${}_{\mathrm{NaCl}}$(m1,wc) and S${}_{\mathrm{NaCl}}$(m2,wc), respectively; Table 2). Although chloride ion binding is still weaker than sodium binding, the structural features of the membrane basically correspond to those of a neutral membrane patch (see above and Table 1).

Analysis of coordination numbers of the ions reveals that the new force-field descriptions enhance the first-shell coordination of chloride ions with lipid tail atoms by about three orders of magnitude, while leaving first-shell coordination of sodium ions with lipid tail atoms essentially unaffected (Table 3). The first-shell coordination number of sodium ions with carbonyl- and phosphate-oxygen atoms is reduced by approximately two orders of magnitude in the new force-field description (Table 3). In particular, the binding to the carbonyl oxygen atoms is reduced in that it occurs almost exclusively in a solvent-separated fashion, as evidenced by almost negligible first-coordination shell peaks (heights of 0.21 and 0.43 for simulations S${}_{\mathrm{NaCl}}$(m1,wc) and S${}_{\mathrm{NaCl}}$(m2,wc), respectively) in comparison to the second-coordination shell peaks (heights of 3.07 and 3.90 for simulations S${}_{\mathrm{NaCl}}$(m1,wc) and S${}_{\mathrm{NaCl}}$(m2,wc), respectively). Corresponding peak heights for simulation S${}_{\mathrm{NaCl}}$(o,wc) are 143.1 and 1.56, respectively, i.e., here, an enhanced direct binding in comparison to solvent-separated binding of sodium ions to the carbonyl oxygen atoms is observed. The force-field dependence of the relative balance of direct- and solvent-separated binding mechanisms of cations to lipid molecules has been noted before [43,44] and is of importance for the parameterization of ions in coarse-grained force-field descriptions (inclusion vs. omission of the first hydration shell in the beads representing ions). Although ion-water Lennard–Jones interaction parameters were not altered, the first-shell ion-water coordination numbers are slightly lower (chloride) or higher (sodium) when the new force-field description is employed (Table 3). Concerning second-shell coordination numbers, similar qualitative trends as for the first shell are observed, except for sodium-ion binding to the carbonyl oxygen atoms, where the new force-field description leads to an enhancement of the second-shell coordination number by about a factor two (Table 3).

**Figure 4:**
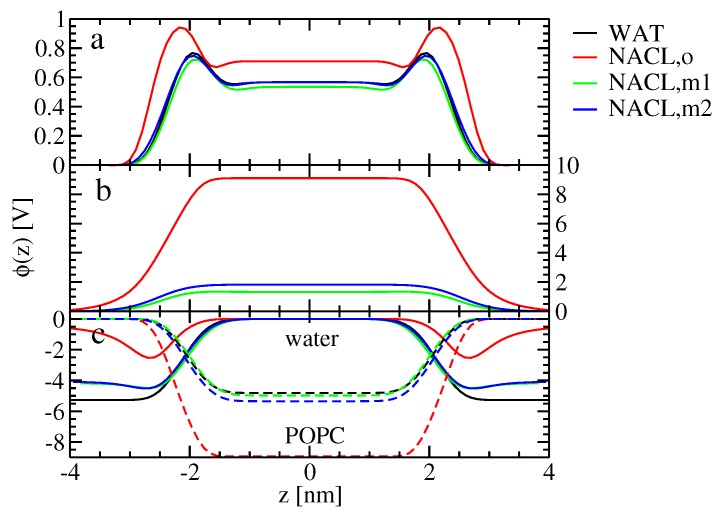
Symmetrized electrostatic potential ϕ(z) along the bilayer normal (Equation (16)), for simulations S${}_{\mathrm{wat}}$(wc), S${}_{\mathrm{NaCl}}$(o,wc), S${}_{\mathrm{NaCl}}$(m1,wc) and S${}_{\mathrm{NaCl}}$(m2,wc), shown as black, red, green and blue curves, respectively. The underlying atom-based charge density $\rho_{a}\left(z\right)$ involves (**a**) all atoms, (**b**) ions or (**c**) only lipid (dashed lines; labeled “POPC”) or water (solid lines; labeled “water”) atoms. The anchoring of the curves was done as described in Section 4.3.1. The simulation acronyms are explained in Table 7. Note that in this illustration, the lipid bilayer is centered.

The alteration of ion-membrane binding is reflected in the electrostatic potential along the bilayer normal. In the absence of ions, the lipid headgroups (with the negatively-charged phosphate groups being closer to the membrane interior than the positively-charged choline groups) lead to a contribution of the electrostatic potential inside the membrane, which is negative with respect to the value in the bulk water (Figure 4c). Water not only dielectrically screens this electrostatic potential contribution, but overcompensates it, such that the electrostatic potential contribution due to water, as well as the total electrostatic potential are positive inside the membrane (Figure 4a,c). It was suggested before that this effect arises from the structure of water at the surface of hydrophobic media [45]; however, this explanation might be misleading because of the limitations of Equation (16) and the fact that preferential water orientation at hydrophobic surfaces is not per se decisive, but must be interpreted relative to a system with isotropically-oriented water molecules [46]. For the old force field, the asymmetric binding of the cations and the anions leads to a contribution of the ions to the electrostatic potential, which is positive inside the membrane (Figure 4b). Both water and lipids dielectrically screen (partially compensate) the effect of the ions on the electrostatic potential, which means that the contribution of both water and lipids to the electrostatic potential inside the membrane (with respect to the water) is decreased compared to the ion-free case. For the new force-field versions showing similar binding for both ion species, the effect of the ions on the electrostatic potential inside the membrane is much smaller. Hence, the change of the total electrostatic potential compared to the ion-free case, as well as the corresponding contributions from water and lipids are much smaller to negligible for the new force-field versions.

Membrane-associated net charge, as (considering apparent ion binding constants; Table 2) strongly present in simulation S${}_{\mathrm{NaCl}}$(o,wc) and nearly vanishing in simulations S${}_{\mathrm{NaCl}}$(m1,wc) and S${}_{\mathrm{NaCl}}$(m2,wc), can cause a separation of membrane bilayer copies. This was already discussed above in terms of structural properties, e.g., an increase in the thickness of the water layer $d_{w}$. However, changes in membrane area or water layer thickness can occur as well via an entirely different mechanism, e.g., because ions interacting with the membrane can push apart or pull closer neighboring lipid molecules. The question about what is the physical mechanism underlying the area-per-lipid increase in the present study can be answered through analyzing the disjoining pressure $P_{\mathrm{TH}}$, which arises from differential ion concentrations in the center of the water layer (Equation (7)). Here, it is found that $P_{\mathrm{TH}}$ is indeed almost increased three-fold with the original force field in comparison to the modified force-field versions, the latter presenting more balanced association of cations and anions with the membrane (Table 4). The disjoining pressure was also calculated as a function of area compressibility, water layer thickness and area-per-lipid change upon ion binding ($P_{\mathrm{MD}}$; Equation (6)). The resulting pressures $P_{\mathrm{MD}}$ obtained from the different simulations suggest that the forces influencing the distance between successive bilayer copies are indeed repulsive when the original force field is employed (positive disjoining pressure; Table 4), but attractive when the modified force-field versions are used (negative disjoining pressure; Table 4). These conclusions are independent of whether the water bilayer thickness and area-per-lipid change upon ion binding are taken from simulations with the weak-coupling or the Parrinello–Rahman barostat (Table 4).

Note that $P_{\mathrm{MD}}$ and $P_{\mathrm{TH}}$ differ by about three orders of magnitude (Table 4). This indicates that the change in area per lipid induced by the new force-field description is actually not due to the alteration of membrane-associated net charge, but must rather be understood as a consequence of ion-specific effects on the arrangement of lipid molecules. Sodium ions can be envisioned as decreasing the area per lipid, because they may be coordinated by oxygen atoms pertaining to multiple lipid molecules at the same time, which decreases the distance between lipid molecules. Because of their more smeared out charge, chloride ions have a somewhat higher affinity to nonpolar membrane regions than sodium ions. The association of chloride ions with more hydrophobic membrane regions leads to a larger exposure of the fatty-acid chains to water, which causes the area per lipid to increase.

**Figure 5:**
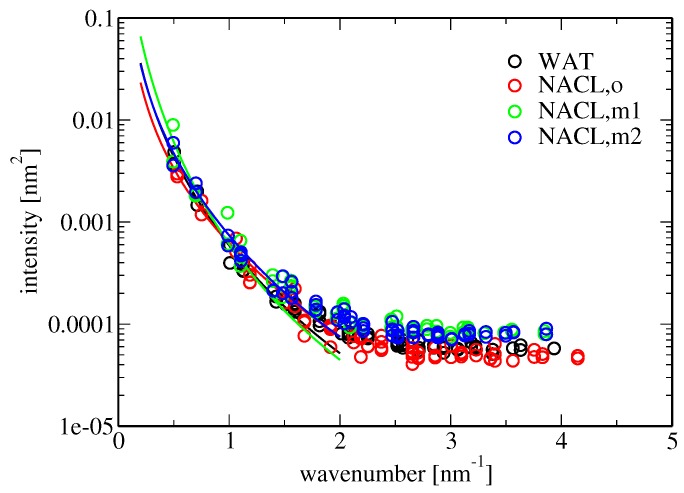
Spectral intensity of membrane undulations $\langle u^{2}\left(q\right)\rangle$ as a function of the wavenumber *q* for simulations L${}_{\mathrm{wat}}$(wc), L${}_{\mathrm{NaCl}}$(o,wc), L${}_{\mathrm{NaCl}}$(m1,wc) and L${}_{\mathrm{NaCl}}$(m2,wc), shown as black, red, green and blue circles, respectively. $\langle u^{2}\left(q\right)\rangle$ was obtained from a Fourier transform of the discretized undulation $\bar{\tilde{z}}\left(x_{i},y_{j}\right)$ (Section 4.3.1). The data refer to the results from the analysis employing twelve grid cells per dimension. Solid lines indicate a fit according to Equation (18), carried out in the low-wavenumber regime. The simulation acronyms are explained in Table 7.

Membrane bending rigidity was calculated according to Equation (18). The results are reported in Table 5. The bending stiffness observed in the simulation involving pure water as the solvent (2.8× $10^{-20}$ J) is similar to those found in comparable simulation studies, e.g., 4× $10^{-20}$ J for the DPPC bilayer studied in [47]. According to experimental investigations, the bending fluctuations of a POPC bilayer increase in the presence of aqueous NaCl (above concentrations of about 0.5 M) [4], and concomitantly, the bending stiffness $k_{c}$ is expected to decrease. Here, the presence of salt in the solvent leads to contradictory results for the change in bending rigidity in comparison to pure water when old or new force-field descriptions are used, namely values of $k_{c}=1.7$, 3.3 or 1.7 $\times \;10^{-20}$ J in simulations L${}_{\mathrm{NaCl}}$(o,wc), L${}_{\mathrm{NaCl}}$(m1,wc) and L${}_{\mathrm{NaCl}}$(m2,wc), respectively (Table 5). Because the error of $k_{c}$ for the salt-free simulation is rather large, it is difficult to assess the effect of membrane-ion binding on $k_{c}$, as well as whether the effect agrees with the experiment. Comparing $k_{c}$ in the presence of salt for the different force fields, the large difference between the similar force-field versions m1 and m2 is striking. This might be due to ill convergence of the simulations or a poor fit to the data points for the spectral intensity of undulation because of an insufficient number of data points in the considered low-wavenumber regime, i.e., a too large grid cell edge length (Equation (18) and Figure 5). Note that the grid employed for the analysis of membrane undulations was rather coarse (Section 4.3.1).

The coefficient *B* (Equation (8)) characterizing leaflet interdigitation is considerably smaller than the value B=1/48 commonly associated with the absence of interdigitation (Table 5), indicating that the two leaflets move in an uncoupled fashion. The underestimation of *B* in comparison to the theoretically-expected value of 1/48 may be due to an underestimation of $k_{c}$ or/and an overestimation of $\kappa_{A}$ or/and of $D_{\mathrm{HH}}$ in the present simulations. For the membrane in pure water, the latter quantities may be compared with readily-available experimental data. Experimentally, $k_{c}$, $\kappa_{A}$ and $D_{\mathrm{HH}}$ are found to be $5\;\times \;10^{-20}$ J [48], 139 kJ·mol${}^{-1}\cdot$nm${}^{-2}$ [49] and 3.83 nm [49], respectively, for a DPPC bilayer at ambient conditions. These values result in B=0.015 (Equation (8)), which is close to B=1/48≈0.02. In comparison to the above experimental data, the simulated membrane shows a bending stiffness that is underestimated by 44% and an area compressibility that is overestimated by 45%, while the bilayer thickness is in fairly good agreement with the experimental value (Table 1 and Table 5). The membrane force field appears to render the membrane too soft (increased susceptibility to bending deformation and lateral compression), which results in the very small *B*-coefficient observed here. Considering the relatively large error in $k_{c}$ (Table 5), the calculated values of *B* are also affected by a rather large statistical uncertainty.

**Figure 6:**
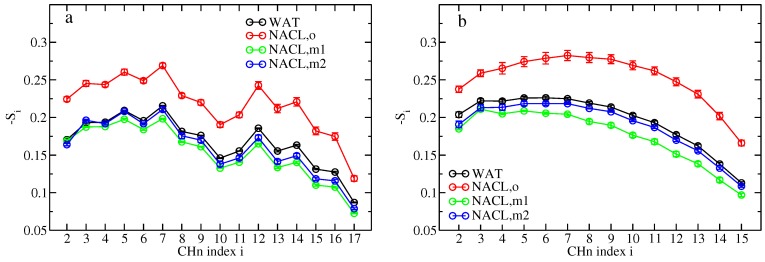
Deuterium order parameter $-S_{i}$ (Equation (11)) of the CH${}_{n}$ groups *i* of the oleoyl (**a**) and palmitoyl (**b**) chains for simulations S${}_{\mathrm{wat}}$(wc), S${}_{\mathrm{NaCl}}$(o,wc), S${}_{\mathrm{NaCl}}$(m1,wc) and S${}_{\mathrm{NaCl}}$(m2,wc), shown as black, red, green and blue circles, respectively. The first (carboxylate) and last CH${}_{n}$ groups are omitted from the analysis. The simulation acronyms are explained in Table 7.

Comparing the effect of ions on lipid ordering and diffusion between the old and new force-field descriptions, the membrane properties appear to be closer to the pure-water situation when the latter is used. Deuterium order parameters of lipid tail CH${}_{n}$ groups experience a minute decrease through sodium and chloride binding in simulations S${}_{\mathrm{NaCl}}$(m1,wc) and S${}_{\mathrm{NaCl}}$(m2,wc) in comparison to simulation S${}_{\mathrm{wat}}$(wc), whereas they are elevated on average by 30.5% for the oleoyl chains and by 30.3% for the palmitoyl chains in simulation S${}_{\mathrm{NaCl}}$(o,wc), i.e., lipid tail order is much enhanced through the unbalanced binding of sodium and chloride ions as occurring with the old force-field description (Figure 6). Similarly, the lipid center of mass diffusion coefficients (across all three examined time scales) are closer to the pure-water situation with the new force-field description in comparison to the old one (Table 6). With the latter, the long, medium and short time scale diffusion coefficients are reduced by 46.2, 31.7 and 13.0%, respectively, in comparison to the the diffusion coefficients found for the membrane in pure water. Thus, the excess of sodium-ion binding in simulation S${}_{\mathrm{NaCl}}$(o,wc) appears to lead to a drastic slowing down of the long time scale lipid motion (Table 6).

Neutron scattering experiments deliver diffusion coefficients on a short timescale of 1–10 ps [50,51]; however, the influence of salts is unknown to date.

Experimental data based on fluorescence correlation spectroscopy (FCS) show that on millisecond timescales, lipid diffusion decreases with increasing NaCl concentration [19]. Such timescales are not routinely accessed by MD simulations. Simulations by Böckmann et al. [19] showed a decrease in lipid diffusion upon the addition of NaCl on nanosecond timescales. When inferring diffusion constants both from the simulations, as well as the experiments, analyses were performed based on a normal diffusion model. However, in fact, lipids undergo anomalous diffusion [52]. Therefore, diffusion constants inferred assuming normal diffusion are effective diffusion constants, which depend on the timescale considered. Table 6 shows that the effective diffusion constants decrease with increasing timescale. The smallest timescales considered in the table (2–10 ps) are similar to the timescales accessed by neutron scattering experiments (1–10 ps). Interestingly, the simulated diffusion coefficients on the 2–10-ps timescale are more similar to experimental values obtained at elevated temperatures ([50] reports a translational diffusion coefficient of $14\;\times \;10^{-8}$ cm${}^{2}\cdot$s${}^{-1}$ for a system at 30 ${}^{\circ }$C and 400 × $10^{-8}$ cm${}^{2}\cdot$s${}^{-1}$ for a system at 63 ${}^{\circ }$C; [51] reports in-plane diffusion coefficients on the order of 15 × $10^{-8}$–600 × $10^{-8}$ cm${}^{2}\cdot$s${}^{-1}$ for a system at 60 ${}^{\circ }$C). As diffusion constants inferred from experiments and simulations typically depend on the timescale considered, also the effect of salt on the diffusion constants may depend on the timescales. Though the FCS experiments show a decrease in lipid diffusion on millisecond timescales [19], to our knowledge, no experimental data on the influence of ions on the effective diffusion constants on (sub)nanosecond timescales are available. On such timescales, the old force field also employed in [19] shows a decrease in lipid diffusion, whereas the force fields with the more realistic ion-membrane binding behavior introduced here do not show a substantial salt effect on lipid diffusion.

## 4. Computational Details

### 4.1. MD Simulations

All simulations were performed with the gromacs 4.6.5 engine [53,54]. Five different systems were simulated: (i) a 1 M aqueous NaCl solution consisting of 121 Na${}^{+}$ ions, 121 Cl${}^{-}$ ions and 6715 water molecules (NACL); (ii) a (small) hydrated bilayer consisting of 8×8 POPC molecules per leaflet and 5120 water molecules (S${}_{\mathrm{wat}}$); (iii) a (small) bilayer consisting of 8×8 POPC molecules per leaflet solvated in a NaCl solution of 50 Na${}^{+}$ ions, 50 Cl${}^{-}$ ions and 5020 water molecules (S${}_{\mathrm{NaCl}}$); (iv) a (large) hydrated bilayer consisting of 16×16 POPC molecules per leaflet and 20480 water molecules (L${}_{\mathrm{wat}}$); (v) a (large) bilayer consisting of 16×16 POPC molecules per leaflet solvated in a NaCl solution of 200 Na${}^{+}$ ions, 200 Cl${}^{-}$ ions and 20,080 water molecules (L${}_{\mathrm{NaCl}}$). The concentration of the NaCl solution in the systems containing the POPC bilayer is about 0.5 M. Those systems were simulated using different force fields (for ion-ion and ion-lipid interactions), barostatting schemes and simulation times. An overview is provided in Table 7.

The simulations were performed under periodic boundary conditions at constant temperature (300 K) and pressure (1 bar). If not specified differently, thermostatting and barostatting were performed using the weak-coupling scheme [55] with coupling times of 0.1 ps and 1 ps, respectively, as well as an isothermal compressibility appropriate for water (4.5 × $10^{-5}$ bar${}^{-1}$) for the barostat. The membrane and solvent (water and, if present, ions) components were separately coupled to the temperature bath. The barostatting was applied semi-isotropically, except for the simulations of the aqueous NaCl solution, where the simulation box was scaled isotropically. The Linear Constraint Solver (LINCS) algorithm [57] was used to constrain all bond lengths to their reference values. The equations of motion were integrated according to the leap-frog algorithm [58], with a step size of 0.002 ps.

A buffered (Verlet) pair list was created based on a maximum allowed error for pair interactions per particle of 0.005 kJ·mol${}^{-1}\cdot$ps${}^{-1}$. It was updated every five time steps. Van der Waals interactions were modeled with the Lennard–Jones potential, truncated at a distance of 1.2 nm, with the potential being shifted such that the energy vanishes at the truncation distance. Electrostatic interactions were computed using the particle-mesh-Ewald algorithm [59], based on a real-space cutoff of 1.2 nm, a grid spacing of 0.12 nm and a fourth order interpolation scheme.

Except for simulations involving the Parrinello–Rahman barostat [56] and for simulations of the NaCl solution, all simulations started from an energy-minimized conformation of a POPC bilayer immersed in solvent and were run for 210 ns, of which the first 90 ns were discarded for equilibration (Table 7). The simulation of the NaCl solution was equilibrated for 1 ns and was simulated for 9 ns for production. Simulations with the Parrinello–Rahman barostat [56] were only simulated for 120 ns, with the initial structure taken as the configuration obtained after 120 ns from the corresponding simulation with the weak-coupling barostat [55]. From these 120 ns, the first 30 ns were discarded for equilibration (Table 7).

For all simulations, except L${}_{\mathrm{wat}}$(wc), L${}_{\mathrm{NaCl}}$(o,wc), L${}_{\mathrm{NaCl}}$(m1,wc) and L${}_{\mathrm{NaCl}}$(m2,wc), coordinates and energies were written to file every 2 ps. For the latter systems, coordinates and energies were written to file every 8 and 2 ps, respectively.

### 4.2. Force Field

Three different force-field parameterizations were employed that differ in the Lennard–Jones interactions between sodium and chloride ions, as well as between those ions and POPC molecules. First, an “unmodified” force-field parameter set that was also used in previous studies [5,14,15,19,36] was employed (simulations S${}_{\mathrm{wat}}$(wc), S${}_{\mathrm{wat}}$(pr), L${}_{\mathrm{wat}}$(wc), S${}_{\mathrm{NaCl}}$(o,wc), S${}_{\mathrm{NaCl}}$(o,pr) and L${}_{\mathrm{NaCl}}$(o,wc); Table 7). This parameter set involves Berger lipid parameters [60], Groningen Molecular Simulation 87 (GROMOS87) parameters for sodium and chloride ions, which are equivalent to these parameters in the GROMOS 43a1 force field [61], and the Simple Point Charge (SPC) water model [62], along with application of the geometric-mean combination rule for the Lennard–Jones $C_{6}$ and $C_{12}$ parameters.

Two modified versions of this force field (indicated with “m1” and “m2” in Table 7) were created to investigate the effect of altered ion-lipid binding on membrane structural, mechanical, dynamic and electrostatic properties. The common features of these two parameter sets are: (i) an increase of the Lennard–Jones $C_{12}$ coefficient for Na${}^{+}$ and Cl${}^{-}$ interactions by a factor $s_{\mathrm{NaCl}}=1.33$ to reproduce the same number of chloride ions (within the distance given by the first minimum in the sodium-chloride radial distribution function) around a sodium ion as observed by Hess and van der Vegt [63] for a 1 M aqueous NaCl solution involving the Kirkwood–Buff force field of Weerasinghe and Smith [64] (indicated as $n_{\mathrm{CIP}}=0.1$ in [63]); (ii) a decrease of the Lennard–Jones $C_{12}$ coefficient for Cl${}^{-}$ interactions with the lipid tail atoms (atom types LP2, LP3 and LH1 in the Berger force field) by a factor $s_{{\mathrm{Cl}}^{-}}=0.057$; (iii) an increase of the Lennard–Jones $C_{12}$ coefficient for Na${}^{+}$ interactions with all lipid atoms by a factor $s_{{\mathrm{Na}}^{+}}$. Versions m1 and m2 differ in the choice for $s_{{\mathrm{Na}}^{+}}$, which was set to 3.2 or 3.0, respectively. Thus, the force-field modifications imply a circumvention of combination rules for the above-mentioned atom pairs. Note that these force-field modifications were not done with the aim to quantitatively reproduce the experimental apparent ion-membrane binding constants, which were determined as [14] 0.437±0.046 M${}^{-1}$ for Na${}^{+}$ ions and 0.401±0.038 M${}^{-1}$ for Cl${}^{-}$ ions for POPC vesicles in a 100 mM aqueous NaCl solution. Rather, it was attempted to achieve approximately similar Na${}^{+}$ and Cl${}^{-}$ binding and to investigate the effect on membrane properties.

### 4.3. Analysis of Simulations

#### 4.3.1. Structural, Mechanical, Dynamic and Electrostatic Membrane Properties

Area per lipid, bilayer thickness, area compressibility and membrane bending rigidity were calculated. Note, in the following, that the POPC membrane lies parallel to the xy-plane of the coordinate system. Prior to any analysis, the membrane was centered around the origin of the computational box. The area per lipid $a_{L}$ is given as:

(9) $$a_{L}=\frac{\langle L_{x}L_{y}\rangle }{N_{L}/2},$$

where $L_{x}$ and $L_{y}$ are the box-edge lengths along the *x* and *y* dimensions and $N_{L}$ is the number of lipid molecules in the total bilayer (Table 7). The angular brackets denote arithmetic averaging over simulation frames. The bilayer thickness $D_{\mathrm{HH}}$ is obtained by measuring the distance between maxima in the density profile of all phosphorus atoms along the *z*-axis. In combination with the average box edge length along the *z*-dimension, $\langle L_{z}\rangle$, the water layer thickness $d_{w}$ is obtained as:

(10) $$d_{w}=\langle L_{z}\rangle -D_{\mathrm{HH}}\;$$

and used in the calculation of the disjoining pressure $P_{\mathrm{MD}}$ (Equation (6)). Here, the quantity $a_{L,o}$ was set to the area per lipid observed in a simulation without ions (S${}_{\mathrm{wat}}$(wc)). The calculation of the disjoining pressure according to Equation (7) involves concentrations $\rho_{{\mathrm{Na}}^{+}}\left(z_{m}\right)$ and $\rho_{{\mathrm{Cl}}^{-}}\left(z_{m}\right)$ in the center of the solution moiety of the system, $z_{m}$. These concentrations were read from the corresponding ion density profiles along the *z*-axis of the computational box. The density profiles were constructed using 500 bins along the *z*-axis, which amounts to bin widths of approximately 0.016–0.018 nm.

Deuterium order parameters $S_{i}$ for CH${}_{n}$ groups *i* in the fatty acid tails were calculated. They are defined as:

(11) $$S_{i}=\frac{1}{2}\langle 3\cos^{2}\theta_{i}-1\rangle ,$$

where $\theta_{i}$ is the angle between the bilayer normal and the C–D bond vector associated with the carbon atom in CH${}_{n}$ group *i*. The angular brackets imply averaging over both lipid molecules and trajectory frames. As the Berger lipid force field [60] uses a united-atom description of the fatty acid tails, the positions of the deuterium atoms were reconstructed based on the appropriate bond hybridization geometries.

Radial distribution functions $g_{ij}\left(r\right)$ between ions *i* and other atoms *j* in the system were calculated as:

(12) $$g_{ij}\left(r\right)=\frac{\langle N_{j}\left(r\right)\rangle }{4\pi r^{2}\Delta r\rho_{j}},$$

where $N_{j}\left(r\right)$ is the number of particles *j* in a shell of width Δr located at distance *r* from particle *i* and $\rho_{j}$ is the number density of particles *j*. Here, sodium or chloride ions were considered as particles *i*, and water oxygen atoms, lipid tail carbon atoms, lipid ester carbonyl oxygen atoms, or lipid ester phosphate oxygen atoms were considered as particles *j*. The number of particles *j* within the first coordination shell of particles *i* is:

(13) $$N_{ij}^{\left(1\right)}=4\pi \rho_{j}\int_{0}^{R_{1}}dr\;r^{2}g_{ij}\left(r\right),$$

where $R_{1}$ indicates the position of the first minimum of $g_{ij}\left(r\right)$. The number of particles *j* within the second coordination shell of particles *i* is:

(14) $$N_{ij}^{\left(2\right)}=-N_{ij}^{\left(1\right)}+4\pi \rho_{j}\int_{0}^{R_{2}}dr\;r^{2}g_{ij}\left(r\right),$$

where $R_{2}$ indicates the position of the second minimum of $g_{ij}\left(r\right)$. The bin width Δr was set to 0.002 nm.

Diffusion coefficients *D* for the center of mass of lipid molecules were calculated based on the Einstein relation:

(15) $$D=\frac{1}{2n}\frac{d}{dt}\langle {\left[r\left(t\right)-r\left(t_{o}\right)\right]}^{2}\rangle ,$$

where n=2 for two-dimensional diffusion of the lipid molecules in the bilayer plane and $\langle {\left[r\left(t\right)-r\left(t_{o}\right)\right]}^{2}\rangle$ is the mean square displacement of the center of mass of lipid molecules at time $t-t_{o}$. Here, the angular brackets imply averaging over lipid molecules and time origins $t_{o}$. In practice, diffusion coefficients were calculated separately for the two monolayers and successively averaged. An error estimate was obtained from the propagation of uncertainty in $\frac{d}{dt}\langle {\left[r\left(t\right)-r\left(t_{o}\right)\right]}^{2}\rangle$ for each monolayer. Furthermore, because lipid diffusion in bilayers is anomalous [52], three time scales (2–6 ns, 100–500 ps and 2–10 ps) in which the lipid molecules show different diffusion behavior (i.e., a plot of mean square displacement vs. time has different slopes) were analyzed.

The (lattice-sum) electrostatic potential along the bilayer normal, ϕ(z), was calculated by double integration of the atom-based charge density $\rho_{a}\left(z\right)$ (see, e.g., [45]):

(16) $$\phi \left(z\right)=C-\epsilon_{o}^{-1}\int_{0}^{\langle L_{z}\rangle /2}dz^{\prime }\;\int_{z^{\prime }}^{\langle L_{z}\rangle /2}dz^{\prime \prime }\;\rho_{a}\left(z^{\prime \prime }\right),$$

where $\epsilon_{o}$ is the permittivity of vacuum and *C* an arbitrary integration constant. In practice, the integration was done numerically based on a histogram of the atom-based charge density, with a bin width of about 0.093 nm. Different subsets of the system were considered as contributing to $\rho_{a}\left(z\right)$. The integration constant *C* was set such that ϕ(z) is zero in the bulk when $\rho_{a}\left(z\right)$ involved all atoms, only ions or only lipid atoms and is zero at the bilayer center when $\rho_{a}\left(z\right)$ involved only water atoms. The electrostatic potential was symmetrized with respect to the bilayer center. Note that the summation over atoms in Equation (16) may be considered inappropriate [65,66], but it was used here in the absence of an unambiguous definition for “molecule-based” summation involving lipid molecules.

Membrane area compressibility $\kappa_{A}$ is calculated according to Equation (4), which, using $a_{L}$ in Equation (9), may be rewritten:

(17) $$\kappa_{A}=\frac{\beta^{-1}a_{L}}{\left(N_{L}/2\right)\delta_{a_{L}}^{2}}.$$

Note that this compressibility is affected by membrane undulations. Hence, it is an effective compressibility that has contributions from the true (intrinsic) area compressibility describing a change in $a_{L}$ along the membrane surface that may actually be curved due to undulations and a second contribution, because here, an area-projection into the xy-plane is analyzed, considering that $a_{L}$ is given by Equation (9) [67]. Because the fluctuations of the area per lipid $\delta_{a_{L}}^{2}$ are spurious in simulations involving the weak-coupling barostat, the Parrinello–Rahman barostat was used for simulations from which $\kappa_{A}$ was determined (simulations S${}_{\mathrm{wat}}$(pr), S${}_{\mathrm{NaCl}}$(o,pr), S${}_{\mathrm{NaCl}}$(m1,pr) and S${}_{\mathrm{NaCl}}$(m2,pr); Table 7).

Simulations of a membrane patch four-times the size of the original membrane (simulations L${}_{\mathrm{wat}}$(wc), L${}_{\mathrm{NaCl}}$(o,wc), L${}_{\mathrm{NaCl}}$(m1,wc) and L${}_{\mathrm{NaCl}}$(m2,wc); Table 7) were performed to calculate the bending stiffness. Specifically, the bending stiffness $k_{c}$ was determined from a fit of [47]:

(18) $$\langle u^{2}\left(q\right)\rangle =\frac{\beta^{-1}}{{\langle L_{x}\rangle }^{2}}{\left(k_{c}q^{4}+\tilde{\gamma }q^{2}\right)}^{-1}$$

to the spectral intensity of membrane undulations $\langle u^{2}\left(q\right)\rangle$ as a function of the wavenumber *q*, where the microscopic surface tension coefficient $\tilde{\gamma }$ also results from the fit. The fit was carried out in the low-wavenumber regime [47], here up to q=2.0 nm${}^{-1}$. The correlation coefficients of the fits were 0.99, 0.91, 0.93 and 0.97 for simulations L${}_{\mathrm{wat}}$(wc), L${}_{\mathrm{NaCl}}$(o,wc), L${}_{\mathrm{NaCl}}$(m1,wc) and L${}_{\mathrm{NaCl}}$(m2,wc), respectively. The undulation intensity $\langle u^{2}\left(q\right)\rangle$ was obtained as described by Lindahl and Edholm [47]. In detail, here, the carbon atoms connecting the phosphoglycerol headgroup and the fatty-acid tails were used to analyze membrane undulation. A shifted set of *z*-coordinates ($\left\{{\tilde{z}}_{i}\right\}$) of those atoms was created by subtracting the arithmetic mean of the *z*-coordinates of the subset of these atoms located in the same bilayer, i.e., ${\tilde{z}}_{i}=z_{i}-N_{a}^{-1}\sum_{j=1}^{N_{a}}z_{j}$, where $z_{i}$ is the *z*-coordinate of a considered carbon atom *i* and the sum runs over all $N_{a}$ of the considered carbon atoms *j* that are in the same bilayer leaflet as atom *i* (including atom *i*). The considered carbon atoms were assigned to two-dimensional histograms according to their *x*- and *y*-coordinates, i.e., the histogram was constructed parallel to the xy-plane. Twelve grid cells per dimension were chosen, corresponding to grid cell edges of about 1.0–1.1 nm, i.e., grid cell areas of 1.0–1.2 nm${}^{2}$ which, considering the observed area per lipids, cover 1–2 lipid molecules. Per frame, for each grid cell $x_{i},y_{j}$, an average $\bar{\tilde{z}}\left(x_{i},y_{j}\right)$ of ${\tilde{z}}_{i}$ over all atoms located in the respective grid cell was calculated. If no atoms were situated in a given cell in a given simulation frame, a value $\bar{\tilde{z}}\left(x_{i},y_{j}\right)$ for the respective cell was determined as the average of $\bar{\tilde{z}}\left(x_{k},y_{l}\right)$ values pertaining to the eight neighboring grid cells (under the consideration of periodic boundary conditions along the *x*- and *y*-dimensions of the computational box). The resulting discrete undulation function $\bar{\tilde{z}}\left(x_{i},y_{j}\right)$ was transformed to reciprocal space by means of the Fastest Fourier Transform in the West 3 (FFTW3) library [68], and the resulting average spectral intensity $\langle u^{2}\left(q\right)\rangle$ per wavenumber was obtained from averaging over all simulation frames.

Whenever time series of the quantity of interest were available, statistical errors were assessed via block averaging [69]. Otherwise, statistical errors were estimated as the standard error pertaining to two samples from dividing a total simulation into two halves (i.e., the standard deviation divided by the square root of two), or from the propagation of uncertainties.

#### 4.3.2. Thermodynamics of Ion-Membrane Binding

Apparent binding constants of sodium and chloride ions to the POPC bilayer were calculated according to Equation (1). This requires knowledge of the number of membrane-bound ion species and of the mean salt concentration in the bulk. The former was obtained as:

(19) $${\tilde{N}}_{b}\left({\mathrm{Na}}^{+}\right)=\int_{0}^{z_{s}}dz\;\rho_{{\mathrm{Na}}^{+}}\left(z\right)a_{L}N_{L}/2\;$$

for sodium ions and, equivalently, as:

(20) $${\tilde{N}}_{b}\left({\mathrm{Cl}}^{-}\right)=\int_{0}^{z_{s}}dz\;\rho_{{\mathrm{Cl}}^{-}}\left(z\right)a_{L}N_{L}/2\;$$

for chloride ions, where $\rho_{{\mathrm{Na}}^{+}}\left(z\right)$ and $\rho_{{\mathrm{Cl}}^{-}}\left(z\right)$ are the number densities of sodium and chloride ions, respectively, along the *z*-axis of the system and $z_{s}$ is the location of the shear plane, defined as that distance along the *z*-axis where the water oxygen number density is equal to half of its bulk value. This choice is suggested from the profile of the average velocity of matter parallel to a charged bilayer exposed to an external electric field parallel to the bilayer [45]. ${\tilde{N}}_{b}\left({\mathrm{Na}}^{+}\right)$ and ${\tilde{N}}_{b}\left({\mathrm{Cl}}^{-}\right)$ differ from the corresponding quantities in Equation (1) in that they refer to only one of the two bilayer leaflets. Therefore, the integration in Equations (19) and (20) can in principle be performed for the two leaflets of the bilayer, giving rise to two values for each of ${\tilde{N}}_{b}\left({\mathrm{Na}}^{+}\right)$ and ${\tilde{N}}_{b}\left({\mathrm{Cl}}^{-}\right)$, which, due to symmetry, are expected to be equal within the statistical error. Note that if ${\tilde{N}}_{b}\left({\mathrm{Na}}^{+}\right)$ and ${\tilde{N}}_{b}\left({\mathrm{Cl}}^{-}\right)$ are used in Equation (1), α(I) has to be calculated as $\tilde{\alpha }\left(I\right)=\frac{{\tilde{N}}_{b}\left(I\right)}{N_{L}/2}$, and:

(21) $$K_{\mathrm{app}}\left(I\right)={\left({\bar{c}}_{\mathrm{blk}}\right)}^{-1}\frac{\tilde{\alpha }\left(I\right)}{1-\tilde{\alpha }\left(I\right)}.\;$$

The mean molar salt concentration in the bulk ${\bar{c}}_{\mathrm{blk}}$ corresponds to the geometric mean of $\rho_{{\mathrm{Na}}^{+}}\left(z_{m}\right)$ and $\rho_{{\mathrm{Cl}}^{-}}\left(z_{m}\right)$, where $z_{m}$ is the center of the water layer along the *z*-axis, in units of moles per liter, i.e., assuming $\rho_{{\mathrm{Na}}^{+}}\left(z\right)$ and $\rho_{{\mathrm{Cl}}^{-}}\left(z\right)$ to be given in units of nm${}^{-3}$, ${\bar{c}}_{\mathrm{blk}}$ reads:

(22) $${\bar{c}}_{\mathrm{blk}}=10^{24}N_{A}^{-1}{\left(\rho_{{\mathrm{Na}}^{+}}\left(z_{m}\right)\rho_{{\mathrm{Cl}}^{-}}\left(z_{m}\right)\right)}^{1/2},$$

where $N_{A}$ is Avogadro’s constant.

## 5. Conclusions

The strength of ion-membrane binding is governed by the equilibrium between solvation and the membrane-interaction of the ions and was not accurately reproduced by the lipid force field from Berger et al. [60] in combination with the SPC water model [62] and GROMOS87 Lennard–Jones parameters for sodium and chloride ions [61]. This might be due to force field shortcomings associated with: (i) the use of ad hoc combination rules for the calculation of heteroatomic Lennard–Jones interaction parameters based on homoatomic ones, which was seen to have a spurious impact on ion-ion interactions; (ii) the use of an ad hoc 12th power of interatomic separation to describe Pauli repulsion in the Lennard–Jones potential, which might be inappropriate for very small ions exerting a large electric field on the surroundings (see also [70]); (iii) the lack of explicit polarization (implicit polarization is, to a certain extent, only accounted for through the permanent molecular dipole moment of the SPC water model), which might be especially important for the nonpolar lipid tail carbon atoms [71] owing to the very high polarizability of alkanes [72].

In the present study, the first problem was addressed through the introduction of scaling factors for the Lennard–Jones $C_{12}$ parameter of ion-lipid and ion-ion interactions. The resulting modified force-field description reproduces better the experimental binding constants for the association of sodium and chloride ions with POPC bilayers, as well as the structural properties of a POPC bilayer in an aqueous sodium-chloride solution. The latter are comparable to salt-free conditions. However, ion aggregation at the bilayer-solution interface still occurs, indicating that points (ii) and (iii) above provide opportunities for future force-field improvement.

## Figures and Tables

**Table 1:** Area per lipid $a_{L}$ (Equation (9)), area compressibility $\kappa_{A}$ (Equation (17)), bilayer thickness $D_{\mathrm{HH}}$ (Section 4.3.1) and water layer thickness $d_{w}$ (Equation (10)) for systems containing membrane patches of 64 lipids per leaflet. The simulation acronyms are explained in Table 7. The area compressibility is only reported for simulations involving the Parrinello–Rahman barostat.

Simulation	$a_{L}$	$\kappa_{A}$	$D_{\mathrm{HH}}$	$d_{w}$
	(nm${}^{2}$)	(kJ·mol${}^{-1}\cdot$nm${}^{-2}$)	(nm)	(nm)
S${}_{\mathrm{wat}}$(wc)	$0.607\pm 0.002{\;}^{a}$	-	3.98	4.13
S${}_{\mathrm{wat}}$(pr)	$0.608\pm 0.002{\;}^{a}$	$202\pm 81{\;}^{b}$	4.19	3.91
S${}_{\mathrm{NaCl}}$(o,wc)	$0.542\pm 0.002{\;}^{a}$	-	4.31	4.71
S${}_{\mathrm{NaCl}}$(o,pr)	$0.542\pm 0.001{\;}^{a}$	$376\pm 84{\;}^{b}$	4.20	4.82
S${}_{\mathrm{NaCl}}$(m1,wc)	$0.629\pm 0.001{\;}^{a}$	-	3.86	3.90
S${}_{\mathrm{NaCl}}$(m1,pr)	$0.623\pm 0.002{\;}^{a}$	$222\pm 24{\;}^{b}$	4.03	3.81
S${}_{\mathrm{NaCl}}$(m2,wc)	$0.617\pm 0.002{\;}^{a}$	-	3.95	3.97
S${}_{\mathrm{NaCl}}$(m2,pr)	$0.626\pm 0.001{\;}^{a}$	$263\pm 5{\;}^{b}$	3.75	4.05

${}^{a}$ Error from block averaging; ${}^{b}$ error reported as the standard error from dividing the simulation into two halves.

**Table 2:** Apparent binding constants $K_{\mathrm{app}}$ (Equation (21)) for sodium and chloride ions, computed from ions binding to either leaflet (which is why two values are reported; Section 4.3.2), the corresponding numbers of bound ions ${\tilde{N}}_{b}\left({\mathrm{Na}}^{+}\right)$ and ${\tilde{N}}_{b}\left({\mathrm{Cl}}^{-}\right)$, respectively (Equations (19) and (20)), and mean salt concentration in the bulk ${\bar{c}}_{\mathrm{blk}}$ (Equation (22)). The underlying ion density profiles along the bilayer normal are displayed in Figure 1 (S${}_{\mathrm{NaCl}}$(o,wc)), Figure 2 (S${}_{\mathrm{NaCl}}$(m1,wc)) and Figure 3 (S${}_{\mathrm{NaCl}}$(m2,wc)). The simulation acronyms are explained in Table 7.

Simulation	$K_{\mathrm{app}}\left({\mathrm{Na}}^{+}\right)$		$K_{\mathrm{app}}\left({\mathrm{Cl}}^{-}\right)$		${\tilde{N}}_{b}\left({\mathrm{Na}}^{+}\right)$		${\tilde{N}}_{b}\left({\mathrm{Cl}}^{-}\right)$		${\bar{c}}_{\mathrm{blk}}$
	(M${}^{-1}$)		(M${}^{-1}$)						(M)
S${}_{\mathrm{NaCl}}$(o,wc)	0.71	1.02	0.009	0.012	12.43	16.47	0.20	0.27	0.34
S${}_{\mathrm{NaCl}}$(m1,wc)	0.34	0.36	0.29	0.30	7.74	8.26	6.77	6.96	0.41
S${}_{\mathrm{NaCl}}$(m2,wc)	0.50	0.28	0.39	0.21	10.66	6.53	8.69	4.97	0.40

**Table 3:** Average number of atoms *j* found in the first ($N_{ij}^{\left(1\right)}$; Equation (13)) and second ($N_{ij}^{\left(2\right)}$; Equation (14)) coordination shells of atom *i*, reported for simulations S${}_{\mathrm{NaCl}}$(o,wc), S${}_{\mathrm{NaCl}}$(m1,wc) and S${}_{\mathrm{NaCl}}$(m2,wc). Atoms *i* are Na${}^{+}$ or Cl${}^{-}$ ions, and atoms *j* are water oxygen atoms (OW), lipid tail carbon atoms (CH${}_{n}$), lipid ester carbonyl oxygen atoms (OC), or lipid ester phosphate oxygen atoms (OP). The simulation acronyms are explained in Table 7.

Simulation		S${}_{\mathrm{NaCl}}$(o,wc)	S${}_{\mathrm{NaCl}}$(m1,wc)	S${}_{\mathrm{NaCl}}$(m2,wc)
*i*,*j*				
Na${}^{+}$, OW	$N_{ij}^{\left(1\right)}{\;}^{a}$	3.36 ± 0.05	4.95 ± 0.09	4.97 ± 0.07
	$N_{ij}^{\left(2\right)}{\;}^{b}$	8.8 ± 0.2	12.7 ± 0.4	12.7 ± 0.2
Na${}^{+}$, OC	$N_{ij}^{\left(1\right)}{\;}^{a}$	1.62 ± 0.05	1.1$\times 10^{-2}\pm 0.1\times 10^{-2}$	1.3$\times 10^{-2}\pm 0.1\times 10^{-2}$
	$N_{ij}^{\left(2\right)}{\;}^{b}$	0.35 ± 0.07	0.652 ± 0.006	0.745 ± 0.002
Na${}^{+}$, OP	$N_{ij}^{\left(1\right)}{\;}^{a}$	8.0$\times 10^{-2}\pm 0.9\times 10^{-2}$	$1.03\times 10^{-3}\pm 0.04\times 10^{-3}{\;}^{c}$	$9.8\times 10^{-4}\pm 0.5\times 10^{-4}{\;}^{c}$
	$N_{ij}^{\left(2\right)}{\;}^{b}$	2.25 ± 0.05	0.97 ± 0.01	0.93 ± 0.06
Na${}^{+}$, CH${}_{n}$	$N_{ij}^{\left(1\right)}{\;}^{a}$	2.76 ± 0.07	$3.2\pm 0.4{\;}^{c}$	$2.8\pm 0.4{\;}^{c}$
	$N_{ij}^{\left(2\right)}{\;}^{b}$	2.6 ± 0.2	3.8 ± 0.8	4.4 ± 0.7
Cl${}^{-}$, OW	$N_{ij}^{\left(1\right)}{\;}^{a}$	7.227 ± 0.007	6.1 ± 0.2	6.3 ± 0.1
	$N_{ij}^{\left(2\right)}{\;}^{b}$	21.81 ± 0.05	18.4 ± 0.7	18.2 ± 0.4
Cl${}^{-}$, CH${}_{n}$	$N_{ij}^{\left(1\right)}{\;}^{a}$	$1.0\times 10^{-3}\pm 0.4\times 10^{-3}{\;}^{c}$	$2.0\pm 0.3{\;}^{c}$	$2.0\pm 0.2{\;}^{c}$
	$N_{ij}^{\left(2\right)}{\;}^{b}$	3.7$\times 10^{-2}\pm 0.3\times 10^{-2}$	1.3 ± 0.5	1.3 ± 0.3

${}^{a}$ Error reported as the standard error from dividing the simulation into two halves; ${}^{b}$ error reported as the standard error from dividing the simulation into two halves along with propagation of uncertainty in $N_{ij}^{\left(1\right)}$ according to Equation (14) for the calculation of $N_{ij}^{\left(2\right)}$; ${}^{c}$ no clear first minimum, but a shoulder.

**Table 4:** Disjoining pressure between successive bilayer copies, evaluated via Equation (6) ($P_{\mathrm{MD}}$) and Equation (7) ($P_{\mathrm{TH}}$). The simulation acronyms are explained in Table 7.

Simulation	$P_{\mathrm{MD}}$	$P_{\mathrm{TH}}$
	(kJ·mol${}^{-1}\cdot$nm${}^{-3}$)	
S${}_{\mathrm{NaCl}}$(o,wc)	$8.55{\;}^{a}$ (8.47)${\;}^{b}$	2.74 × $10^{-3}$
S${}_{\mathrm{NaCl}}$(m1,wc)	$-2.06{\;}^{a}$ (−1.44)${\;}^{b}$	1.00 × $10^{-3}$
S${}_{\mathrm{NaCl}}$(m2,wc)	$-1.09{\;}^{a}$ (−1.92)${\;}^{b}$	1.04 × $10^{-3}$

${}^{a}$ Using all quantities in Equation (6) from the indicated simulation, except for $\kappa_{A}$, which was obtained from the corresponding simulation with the Parrinello–Rahman barostat and $a_{L,o}$, which corresponds to simulation S${}_{\mathrm{wat}}$(wc); ${}^{b}$ the value in parentheses does not pertain to the indicated simulation, but to the corresponding simulation with the Parrinello–Rahman barostat (with $a_{L,o}$ being taken from simulation S${}_{\mathrm{wat}}$(pr)).

**Table 5:** Bending stiffness $k_{c}$ (Equation (18)) for simulations of large membrane patches in pure water or a NaCl solution described with the original or modified force-field versions. The average box-edge length along the (quadratic) membrane plane $\langle L_{x}\rangle \left(=\langle L_{y}\rangle \right)$ and the microscopic surface tension coefficient $\tilde{\gamma }$ (Equation (18)) are also provided. The coefficient *B* (Equation (8)) characterizing the extent of membrane interdigitation was calculated using $k_{c}$ reported in this table along with the membrane thickness $D_{\mathrm{HH}}$ and area compressibility $\kappa_{A}$ reported in Table 1 for the corresponding small systems S${}_{\mathrm{wat}}$(pr), S${}_{\mathrm{NaCl}}$(o,pr), S${}_{\mathrm{NaCl}}$(m1,pr) and S${}_{\mathrm{NaCl}}$(m2,pr). The simulation acronyms are explained in Table 7.

Simulation	$k_{c}$	$\langle L_{x}\rangle$	$\tilde{\gamma }$	*B*
	($10^{-20}$ J)	(nm)	(mN·m${}^{-1}$)	($10^{-3}$)
L${}_{\mathrm{wat}}$(wc)	$2.8\pm 0.7{\;}^{a}$	$12.47\pm 0.01{\;}^{b}$	$17.3\pm 1.9{\;}^{a}$	$4.8\pm 2.2{\;}^{c}$
L${}_{\mathrm{NaCl}}$(o,wc)	$1.7\pm 0.2{\;}^{a}$	$11.83\pm 0.01{\;}^{b}$	$31.1\pm 3.5{\;}^{a}$	$1.5\pm 0.4{\;}^{c}$
L${}_{\mathrm{NaCl}}$(m1,wc)	$3.3\pm 0.1{\;}^{a}$	$12.76\pm 0.01{\;}^{b}$	$8.3\pm 0.1{\;}^{a}$	$5.5\pm 0.6{\;}^{c}$
L${}_{\mathrm{NaCl}}$(m2,wc)	$1.7\pm 0.1{\;}^{a}$	$12.70\pm 0.01{\;}^{b}$	$17.6\pm 0.5{\;}^{a}$	$2.8\pm 0.2{\;}^{c}$

${}^{a}$ Error reported as the standard error from dividing the simulation into two halves; ${}^{b}$ error from block averaging; ${}^{c}$ error from propagation of uncertainties in $k_{c}$ and $\kappa_{A}$, neglecting any error in $D_{\mathrm{HH}}$ (values for $\kappa_{A}$ and $D_{\mathrm{HH}}$ from Parrinello–Rahman (pr) simulations of corresponding small systems reported in Table 1).

**Table 6:** Long- (2–6 ns), medium- (100–500 ps) and short-timescale (2–10 ps) lipid center of mass diffusion coefficients (Equation (15)) for simulations S${}_{\mathrm{wat}}$(wc), S${}_{\mathrm{NaCl}}$(o,wc), S${}_{\mathrm{NaCl}}$(m1,wc) and S${}_{\mathrm{NaCl}}$(m2,wc). The time intervals refer to the regime used for a linear fit to the mean square displacement. The simulation acronyms are explained in Table 7.

Time Scale	2–6 ns	100–500 ps	2–10 ps
Simulation	*D* ($10^{-8}$ cm${}^{2}\cdot$s${}^{-1}$)		
S${}_{\mathrm{wat}}$(wc)	7.8 ± 0.3	45.4 ± 0.7	386 ± 3
S${}_{\mathrm{NaCl}}$(o,wc)	4.2 ± 0.2	31.0 ± 0.7	336 ± 3
S${}_{\mathrm{NaCl}}$(m1,wc)	8.1 ± 0.3	46.1 ± 0.4	374 ± 3
S${}_{\mathrm{NaCl}}$(m2,wc)	7.5 ± 0.3	43.4 ± 0.7	366 ± 3

**Table 7:** Overview of the performed simulations. The first column provides an acronym for the different simulations, consisting of a label for the simulated system (NACL, S${}_{\mathrm{wat}}$, S${}_{\mathrm{NaCl}}$, L${}_{\mathrm{wat}}$ or L${}_{\mathrm{NaCl}}$; Section 4.1) and an indication of the employed force field (o, m1, m2) and barostatting (wc, pr). $N_{\mathrm{wat}}$ and $N_{\mathrm{NaCl}}$ denote the number of water molecules and cation-anion ion pairs, respectively; $N_{L}$ denotes the number of lipid molecules. The parameters $s_{{\mathrm{Cl}}^{-}}$, $s_{{\mathrm{Na}}^{+}}$ and $s_{\mathrm{NaCl}}$ specify the scaling factors applied to chloride ion-lipid (tail), sodium ion-lipid and sodium ion-chloride ion $C_{12}$ Lennard–Jones parameters. The total simulation time is reported, along with the length of the initial time period discarded for equilibration in parentheses. Barostatting was done with either the weak-coupling (wc) [55] or the Parrinello–Rahman (pr) [56] barostat. Except for simulation NACL, where barostatting was applied isotropically (iso.), a semi-isotropic (sem. iso.) scaling of box edges was applied.

Simulation	System	$N_{\mathrm{wat}}$	$N_{\mathrm{NaCl}}$	$N_{L}$	$s_{{\mathrm{Cl}}^{-}}$, $s_{{\mathrm{Na}}^{+}}$, $s_{\mathrm{NaCl}}$	Simulation Time (ns)	Barostatting
NACL	NACL	6715	121	0	-, -, 1.33	10 (1)	wc, iso.
S${}_{\mathrm{wat}}$(wc)	S${}_{\mathrm{wat}}$	5120	0	128	1.0, 1.0, 1.0	210 (90)	wc, sem. iso.
S${}_{\mathrm{wat}}$(pr)	S${}_{\mathrm{wat}}$	5120	0	128	1.0, 1.0, 1.0	120 (30)	pr, sem. iso.
L${}_{\mathrm{wat}}$(wc)	L${}_{\mathrm{wat}}$	20,480	0	512	1.0, 1.0, 1.0	210 (90)	wc, sem. iso.
S${}_{\mathrm{NaCl}}$(o,wc)	S${}_{\mathrm{NaCl}}$	5020	50	128	1.0, 1.0, 1.0	210 (90)	wc, sem. iso.
S${}_{\mathrm{NaCl}}$(o,pr)	S${}_{\mathrm{NaCl}}$	5020	50	128	1.0, 1.0, 1.0	120 (30)	pr, sem. iso.
S${}_{\mathrm{NaCl}}$(m1,wc)	S${}_{\mathrm{NaCl}}$	5020	50	128	0.057, 3.2, 1.33	210 (90)	wc, sem. iso.
S${}_{\mathrm{NaCl}}$(m2,wc)	S${}_{\mathrm{NaCl}}$	5020	50	128	0.057, 3.0, 1.33	210 (90)	wc, sem. iso.
S${}_{\mathrm{NaCl}}$(m1,pr)	S${}_{\mathrm{NaCl}}$	5020	50	128	0.057, 3.2, 1.33	120 (30)	pr, sem. iso.
S${}_{\mathrm{NaCl}}$(m2,pr)	S${}_{\mathrm{NaCl}}$	5020	50	128	0.057, 3.0, 1.33	120 (30)	pr, sem. iso.
L${}_{\mathrm{NaCl}}$(o,wc)	L${}_{\mathrm{NaCl}}$	20,080	200	512	1.0, 1.0, 1.0	210 (90)	wc, sem. iso.
L${}_{\mathrm{NaCl}}$(m1,wc)	L${}_{\mathrm{NaCl}}$	20,080	200	512	0.057, 3.2, 1.33	210 (90)	wc, sem. iso.
L${}_{\mathrm{NaCl}}$(m2,wc)	L${}_{\mathrm{NaCl}}$	20,080	200	512	0.057, 3.0, 1.33	210 (90)	wc, sem. iso.
